# An experimental paradigm to manipulate physiological arousal using consecutive successes

**DOI:** 10.1016/j.isci.2025.113961

**Published:** 2025-11-06

**Authors:** Kagari Yamada, Kohei Miyata, Kazutoshi Kudo

**Affiliations:** 1 Graduate School of Arts and Sciences, The University of Tokyo, Tokyo, Japan; 2 Center for Information and Neural Networks (CiNet), National Institute of Information and Communications Technology (NICT), Suita, Japan; 3 Japan Society for the Promotion of Science, Tokyo, Japan; 4 Center for Brain Science, RIKEN, Wako, Japan

**Keywords:** Physiology, Neuroscience, Psychology

## Abstract

This study presents an experimental paradigm using the concept of consecutive successes to examine how performance changes under psychological pressure. In Experiment 1 (*N* = 15), participants performed a force exertion task aiming for 10 consecutive successes, while heart rate and force error were recorded as measures of arousal and task accuracy. Heart rate increased exponentially with the number of consecutive successes, exceeding levels reported in previous studies. This demonstrates that the manipulation induced strong arousal often linked to pressure. Contrary to the expected inverted U-shaped relationship between pressure and performance, performance improved linearly. In Experiment 2 (*N* = 15), we changed the goal to 100 total successes to rule out order effects. The changes in heart rate and performance associated with consecutive successes were absent. This approach offers an efficient and scalable method to induce pressure-like conditions in laboratory settings, providing a useful tool for studying how arousal levels influence performance.

## Highlights

• When the goal is consecutive streaks, consecutive successes induce large heart-rate increases
• Performance in a force task improves linearly with consecutive successes for a streak goal
• No streak-linked arousal or performance change when the goal is totals
• Mediation shows arousal alone does not explain performance gains

## Graphical abstract

**Figure:**
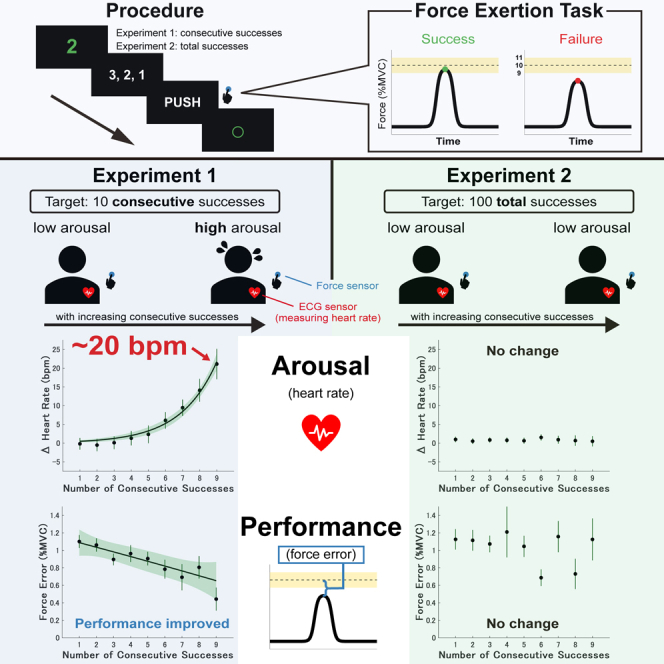

## Introduction

When potential rewards become excessively large, performance may paradoxically decline, a phenomenon known as “choking under pressure.”^1^ This occurs when psychological pressure elevates arousal to a level that impairs task execution. The over-arousal theory, based on the inverted U hypothesis,^2^ posits that performance improves with increasing arousal up to an optimal point, beyond which further arousal leads to deterioration. Although many studies have examined choking under pressure, few have explored how performance changes across a wide range of pressure levels,^3^ partly because most prior research has compared only two conditions: pressure and no pressure. Previous studies have shown that pressure can be induced through various manipulations such as competition,^4^^,^^5^^,^^6^^,^^7^^,^^8^^,^^9^ cooperation,^10^ evaluation by others,^7^^,^^11^^,^^12^^,^^13^^,^^14^^,^^15^^,^^16^ and monetary incentives.^17^^,^^18^^,^^19^^,^^20^ Such binary comparisons limit our understanding of the nuanced relationship between pressure and performance because real-world pressure should not be regarded solely as a binary pattern of present or absent, but rather as a spectrum.^21^ Perhaps due in part to this limitation, findings regarding the relationship between pressure and performance have been inconsistent.^3^ For instance, some studies have identified an inverted U-shaped relationship between monetary incentives and performance, whereas others have not.^17^^,^^18^^,^^19^^,^^20^

To address this gap, we developed an experimental paradigm that progressively increases task difficulty by requiring consecutive successes, thereby indirectly manipulating situations likely to be associated with psychological pressure. Conceptually, psychological pressure is understood as a state that elevates physiological arousal,^22^^,^^23^ which in turn, via sympathetic nervous system activation, can be indexed by measures such as heart rate.^24^^,^^25^ While our manipulation did not allow us to confirm an increase in psychological pressure, our manipulation elicited a substantial increase in heart rate, a physiological marker often associated with pressure.^7^^,^^15^^,^^16^^,^^26^^,^^27^^,^^28^^,^^29^^,^^30^^,^^31^ Because assuming that the success rate is always constant, the probability of additional successes declines exponentially as the number of consecutive successes increases, participants likely experienced escalating task demands, which may have induced greater arousal. In Experiment 1, we observed that heart rate increased as consecutive successes accumulated, while performance also improved—contrary to predictions from the inverted U hypothesis. However, this performance improvement may stem from an order effect driven by the memory of immediately preceding successful trials, rather than from the effects of psychological pressure. Indeed, studies on motor learning suggest that memories of recent performances contribute to short-term adaptation and improve the precision of subsequent actions.^32^^,^^33^ Therefore, it is crucial to disentangle these potential learning effects from the effects of pressure.

To clarify this, Experiment 2 used the same task but altered the participants’ goal from achieving consecutive successes to maximizing total successes, thereby decoupling task structure from pressure awareness. By reducing participants’ focus on streaks, this manipulation allowed us to examine whether the observed changes in heart rate and performance in Experiment 1 could be attributed to pressure or simply to sequence-related performance improvements. This experimental approach enables a more continuous and cost-effective examination of pressure-related effects and offers insight into the complex relationship between physiological arousal and performance.

## Results

### Overview of the experimental paradigm

**Figure 1:**
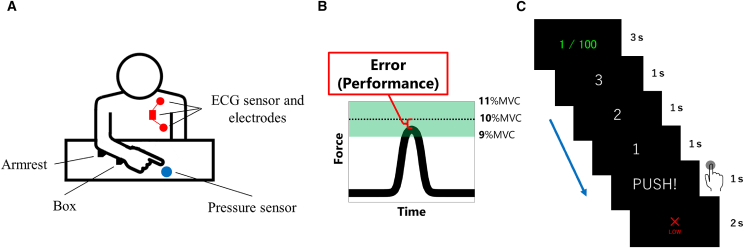
Experimental paradigm and task procedure (A) Experimental setup used in the fingertip force exertion task. Participants placed their index finger on a force sensor to exert a specified amount of force. (B) Example trace of force exertion during a single trial. Participants were instructed to exert 10% of their MVC. A trial was classified as successful if the exerted force fell within ±10% of the target (i.e., between 9% and 11% MVC). The absolute value of the relative error from the 10% MVC target was used as the performance measure. (C) Sequence of events within each trial. First, a screen indicated either the current number of consecutive successes (Experiment 1) or total successes (Experiment 2). After a countdown, participants attempted to exert 10% MVC. Following the response, visual feedback informed the participant of success or failure based on their performance.

Fifteen participants took part in Experiment 1, and a separate group of 15 participants took part in Experiment 2. Participants performed a fingertip force exertion task aiming to reach 10% of their maximal voluntary contraction (MVC) (Figure 1A). Success was defined as exerting force between 9% and 11% of MVC (Figure 1B). In Experiment 1, participants aimed for 10 consecutive successes, while in Experiment 2, the goal was to achieve a total of 100 successes (Figure 1C). Heart rate and absolute force error relative to the 10% MVC target as performance were recorded in each trial.

### Consecutive successes increase heart rate in Experiment 1 but not Experiment 2

To examine how heart rate changed with consecutive successes in Experiment 1, we applied a linear mixed-effects model (LMM). This model included the number of consecutive successes as a fixed effect and included random intercepts and slopes for each participant to account for individual differences in baseline heart rate and reactivity to the manipulation. The model revealed a significant positive linear effect (*β* = 1.82, 95% confidence interval [CI] [0.89, 2.74], *p* < 0.001; Figure S1A) and, crucially, a significant positive quadratic effect (*β* = 0.32, 95% CI [0.20, 0.44], *p* < 0.001; Figure S1B). Furthermore, a likelihood ratio test confirmed that this quadratic model provided a significantly better fit than a linear-only model (*χ*^*2*^ (2) = 80.55, *p* < 0.001). These results indicate that heart rate increased in an accelerating, non-linear fashion as the success streak grew.

**Figure 2:**
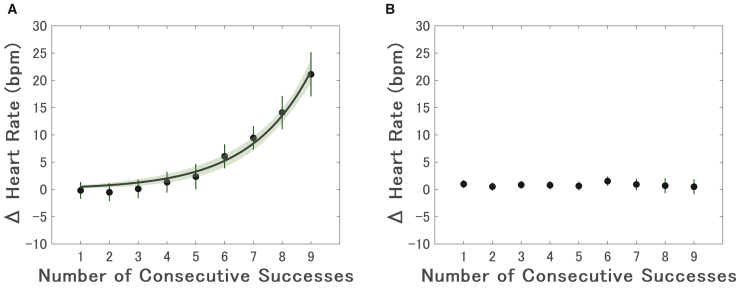
Heart rate difference from the resting value as a function of consecutive successes (A) In Experiment 1, heart rate increased significantly with the number of consecutive successes, indicating elevated physiological arousal associated with performance streaks (*β* = 1.82, 95% CI [0.89, 2.74], *p* < 0.001; LMM). An exponential model was similarly fitted (*R*^*2*^ = 0.98). Shaded areas represent 95% CIs, and vertical bars indicate between-participant standard error. The exponential model provided a better fit than the linear model (*χ*^*2*^ (2) = 80.55, *p* < 0.001; likelihood ratio test), as indicated by a higher coefficient of determination and lower AIC and BIC values (exponential AIC = 2.08, BIC = 2.47; linear AIC = 22.3, BIC = 22.7). (B) Experiment 2 showed no significant relationship between consecutive successes and heart rate (*β* = −0.023, 95% CI [−0.18, 0.14], *p* = 0.77; LMM), suggesting that arousal did not increase when consecutive successes were not emphasized. Vertical bars denote between-participant standard errors.

To further characterize the shape of this accelerating trend at the group-averaged level, we fitted an exponential function to the mean heart rate values (Figure 2A). Consistent with the significant quadratic term in our LMM, the exponential model provided an excellent descriptive fit to the data (coefficient of determination: *R*^*2*^ = 0.98), far exceeding a simple linear model (*R*^*2*^ = 0.85). Although information criteria are best used to compare models of the same class, the lower Akaike information criterion (AIC) and Bayesian information criterion (BIC) values for the exponential model (exponential AIC = 2.08, BIC = 2.47; linear AIC = 22.3, BIC = 22.7) are consistent with the conclusion that an exponential function more accurately describes the accelerating nature of the heart rate increase.

In contrast, Experiment 2 showed no significant relationship between consecutive successes and heart rate (*β* = −0.023, 95% CI [−0.18, 0.14], *p* = 0.77; Figure 2B), suggesting that heart rate did not increase when consecutive successes were not the explicit goal.

### Performance improves with consecutive successes in Experiment 1 but not Experiment 2

**Figure 3:**
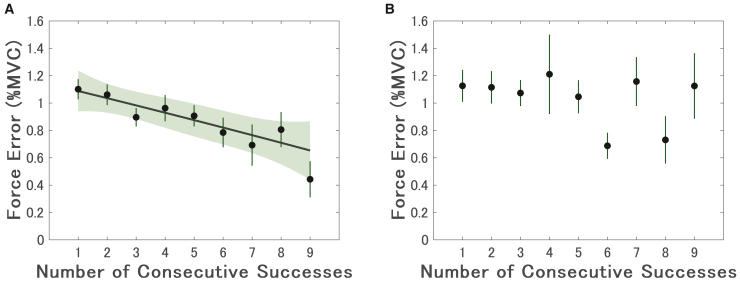
Force error as a function of consecutive successes (A) In Experiment 1, force error decreased significantly with the number of consecutive successes (*β* = −0.054, 95% CI [−0.083, −0.026], *p* < 0.001; LMM), indicating performance improvement associated with performance streaks. (B) In contrast, Experiment 2 showed no significant relationship between consecutive successes and force error (*β* = 0.073, 95% CI [−0.040, 0.19], *p* = 0.19; LMM), suggesting that performance did not improve when consecutive successes were not emphasized. Solid lines represent linear regression fits based on significant fixed-effects coefficients, with shaded areas indicating 95% CIs. Vertical bars denote between-participant standard errors.

Force error decreased significantly as the number of consecutive successes increased in Experiment 1 (*β* = −0.054, 95% CI [−0.083, −0.026], *p* < 0.001; Figure 3A), indicating linear improvement in performance. A quadratic model did not explain performance changes (*β* = −0.000028, 95% CI [−0.0011, 0.0010], *p* = 0.96). In Experiment 2, force error was not significantly predicted by the number of consecutive successes (*β* = 0.073, 95% CI [−0.040, 0.19], *p* = 0.19; Figure 3B), suggesting that performance was unaffected when consecutive successes were not emphasized as a goal.

An independent samples *t* test showed success rates did not differ significantly between Experiment 1 (51.1% ± 10.1%) and Experiment 2 (50.2% ± 10.3%) (*t* (14) = 0.22, *p* = 0.83, Cohen’s *d* = 0.08), indicating comparable task difficulty across experiments.

### Physiological arousal does not mediate the effect of consecutive successes on performance

To test the hypothesis that physiological arousal mediates the relationship between consecutive successes and performance, we conducted a multilevel mediation analysis. The analysis confirmed that the number of consecutive successes significantly predicted an increase in heart rate (β = 1.82, *p* < 0.001), indicating successful arousal induction. However, in the full mediation model, heart rate did not significantly predict force error when controlling for consecutive successes (β = −0.005, *p* = 0.30). A Sobel test confirmed that the indirect effect of consecutive successes on force error via heart rate was not statistically significant (indirect effect = −0.009, z = −1.02, *p* = 0.31). Interestingly, the direct effect of consecutive successes on force error remained significant in the mediation model (β = −0.044, *p* < 0.05), suggesting that performance improvement is driven by factors other than, or in addition to, the increase in heart rate.

## Discussion

This study aimed to conceptualize and reproduce psychological pressure as a continuous spectrum rather than a binary condition, using a consecutive success paradigm. Our central hypothesis was that increasing the number of consecutive successes would elevate psychological pressure, as reflected by physiological arousal, and that this increase in pressure would in turn affect task performance.^2^^,^^23^ Heart rate, used as a proxy for arousal, increased significantly with the number of consecutive successes in Experiment 1, supporting the hypothesis that pressure accumulated incrementally as participants approached longer success streaks and neared the goal of achieving 10 consecutive successes.

**Figure 4:**
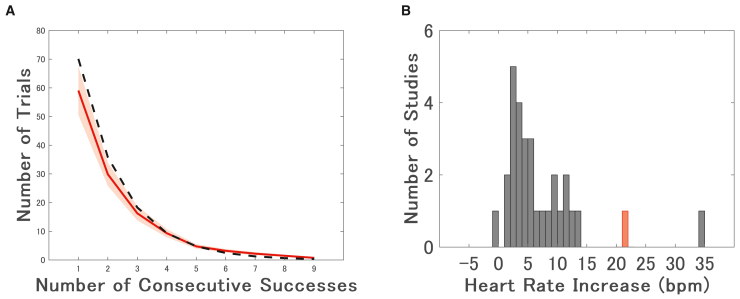
Decrease in the number of trials per participant as consecutive successes increase in Experiment 1 and distribution of heart rate increases reported in previous pressure-induction studies and the present study (A) The red line indicates the actual mean number of trials per participant observed at each level of consecutive success. The shaded area represents the SEM. The black dotted line represents the expected number of trials, estimated based on the average total number of trials per participant (268.6; see Table S2) and the overall success rate in Experiment 1 (51.1%). The divergence between the actual (red) and theoretical (black) lines, particularly at longer streaks, is consistent with our finding that performance improved (i.e., the actual success rate increased) as consecutive successes accumulated. (B) Histogram illustrating the number of studies reporting various magnitudes of heart rate increase (in bpm) following psychological pressure induction. Black bars represent prior studies using categorical manipulations (e.g., incentives, evaluation, time pressure), which typically reported modest increases of 0–10 bpm. In contrast, the red bar represents the current study, which observed increases of up to 20 bpm. This visual comparison highlights the potential of the consecutive success paradigm to elicit stronger physiological arousal than traditional methods. See Table S1 for a list of the prior studies included in this analysis.

Importantly, this heart rate increase followed an exponential trajectory, suggesting a non-linear amplification of arousal with additional consecutive successes. This pattern aligns with the probabilistic structure of the task: as streaks grow, the probability of further success diminishes, making longer success streaks increasingly rare for participants (Figure 4A). Notably, the actual number of trials at longer streaks slightly exceeded the theoretical expectation, a finding consistent with the observed performance improvement during these high-pressure situations. Previous studies have often relied on categorical manipulations (e.g., monetary incentives,^6^^,^^7^^,^^8^^,^^9^^,^^13^^,^^14^^,^^15^^,^^16^^,^^17^^,^^18^^,^^19^^,^^20^^,^^31^^,^^34^^,^^35^^,^^36^^,^^37^^,^^38^ social evaluation,^13^^,^^14^^,^^15^^,^^16^^,^^29^^,^^30^^,^^31^^,^^39^ competition,^4^^,^^5^^,^^6^^,^^7^^,^^8^^,^^9^^,^^31^ heights,^28^^,^^40^^,^^41^ or time constraints^26^^,^^42^) to induce pressure, typically resulting in modest increases in heart rate—most commonly in the range of 0–10 bpm (Figure 4B). In contrast, our consecutive success paradigm elicited increases of up to approximately 20 bpm, substantially exceeding the heart rate changes reported in prior research. Notably, this was achieved without the need for external evaluators, financial rewards, or socially evaluative contexts. This efficiency highlights the strength of the consecutive success approach in inducing substantial pressure in a controlled and scalable manner.

Contrary to the predictions of the inverted U hypothesis,^2^ which suggests that performance should deteriorate at high arousal levels, performance in our study improved linearly with increasing pressure. One plausible explanation is that the task was relatively easy, and therefore the optimal arousal level for peak performance was not exceeded. This interpretation is consistent with recent work suggesting that easier tasks have higher optimal arousal thresholds.^3^^,^^43^ Thus, pressure-induced arousal may have facilitated, rather than hindered, performance.

However, an alternative explanation is that performance improvement was due to order effects or momentum from prior successes^32^^,^^33^ rather than pressure itself. To test this, Experiment 2 decoupled pressure from task structure by shifting participants' target from consecutive to total successes. Despite identical task difficulty across experiments, heart rate no longer increased with the number of consecutive successes and performance did not improve. These findings suggest that awareness of consecutive success—and not mere success repetition—was necessary to elicit both increased arousal and enhanced performance. This dissociation provides strong evidence that performance gains in Experiment 1 were attributable to psychological pressure rather than the order effects. One could further argue that the effects in Experiment 1 were driven not by the streak itself, but by simple proximity to a numerical goal. However, control analyses from Experiment 2 do not support this interpretation. We found that neither heart rate increased nor performance improved as participants approached their goal of 100 total successes (Figures S4A and S4B). These null results suggest that both the escalating arousal and the corresponding performance enhancement are specific to the fragile nature of a consecutive success streak, where any single error leads to a complete reset, rather than mere proximity to a finishing line.

The results of our multilevel mediation analysis provided a more nuanced and unexpected picture of the arousal-performance relationship. While our paradigm successfully induced physiological arousal that increased with consecutive successes, this arousal did not statistically mediate the observed performance improvement. The effect of the consecutive success streak on performance remained strong even after accounting for heart rate changes, suggesting a significant direct effect.

This null mediation finding is informative. It suggests that the link between the pressure-inducing context and performance improvement is more complex than a simple arousal-based mechanism. A plausible explanation is that the ability to translate the heightened state of a success streak into performance gains is heavily dependent on stable individual traits. Prior work has shown that factors like working memory capacity and anxiety levels are crucial in modulating performance under pressure.^17^^,^^18^^,^^44^^,^^45^^,^^46^ It is likely that such traits govern the efficiency of cognitive control during a high-pressure streak, a process that is not fully captured by cardiovascular activity alone. This highlights the complex interplay between the situation, physiological arousal, and stable individual traits in shaping performance and cautions against treating a single physiological measure as a complete proxy for this multifaceted process.

Furthermore, the utility of our paradigm lies in its ability to dynamically and transiently manipulate arousal on a trial-by-trial basis. This feature is not a limitation but a key strength, allowing for the specific investigation of phasic (task-evoked) arousal. This approach contrasts sharply with methods like pharmacological intervention, which induce a more sustained, tonic state of arousal.^47^^,^^48^ This is a critical distinction, as a trade-off has been reported between the tonic and phasic activation of the locus coeruleus, a key nucleus for promoting arousal.^49^^,^^50^ By inducing transient, task-locked arousal, our paradigm provides a unique tool to explore how this phasic mode of arousal impacts performance. In sum, this study provides an effective method for manipulating psychological pressure. Our results, which show a linear performance improvement for a simple task, align with a key finding from the original Yerkes and Dodson (1908) study. This work offers a valuable experimental paradigm for further exploration of pressure-performance dynamics, particularly in how they differ by task difficulty.

### Limitations of the study

Several limitations warrant consideration. First, while heart rate served as a convenient and objective marker of arousal, it cannot unambiguously index psychological pressure. Psychological states other than pressure can also influence physiological indicators such as heart rate.^51^ It is important to note that this constitutes a form of reverse inference.^52^^,^^53^ Future research should incorporate self-report measures of perceived pressure to enhance the reliability of manipulating psychological pressure.

Second, our study exclusively involved male participants. While this was a practical decision related to the experimental procedure, it limits the generalizability of our findings. Future research should include participants of other genders to investigate potential sex-based differences in physiological and performance responses to pressure.

Next, individual differences in pressure sensitivity (e.g., personality traits) were not assessed but may have influenced both heart rate reactivity and performance.^46^ Incorporating such factors could enrich interpretations of how pressure impacts performance across diverse individuals.

In addition, the uneven distribution of trials across levels of consecutive success presents a methodological limitation. As the number of consecutive successes increases, the number of corresponding trials decreases exponentially (Tables S2 and S3), introducing potential biases and limiting statistical power at higher levels. While analyses restricted to participants who achieved at least 10 successes yielded consistent results (Figure S2), future work should adopt trial-balancing or hierarchical modeling approaches to mitigate such skews.

This issue of data sparsity also relates to another limitation concerning the peak level of arousal induced and its relation to the inverted U hypothesis. It is possible that setting a higher goal—for instance, 15 consecutive successes—could have pushed participants further along the arousal curve, potentially revealing a performance downturn. However, our choice of a 10-success goal was a deliberate trade-off. Given that not all participants achieved this already difficult goal, a higher target would have severely exacerbated the problem of sparse data at high streak counts and risked undermining participant motivation. While the levels of arousal achieved in our study already substantially exceed those typically reported in pressure-induction research, developing novel paradigms that can robustly and ethically probe performance at these extreme levels of arousal remains an important challenge for future research.

Furthermore, modeling the interaction between pressure and order effects remains an open challenge. Although Experiment 2 helped to control for main effects of task order, interactions between trial sequence and pressure manipulation were not directly tested. Future work should explicitly incorporate these interactions into statistical models.

A further limitation is that our study, by design, cannot establish a definitive causal link between the observed physiological arousal and performance improvement. While our paradigm effectively induced arousal, this arousal is inherently tied to the psychological context of the success streak. To causally test the specific role of arousal, it would be necessary to manipulate it independently from the task context. Future studies could address this limitation by combining our psychological paradigm with non-invasive neurostimulation techniques, such as auricular vagus nerve stimulation.^54^^,^^55^^,^^56^ Such a dual-intervention approach would enable the orthogonal manipulation of arousal through both psychological and physiological pathways, making it possible to isolate the causal impact of arousal itself on performance under pressure.

Finally, we acknowledge that the overall sample size in this study was relatively small. To evaluate whether the observed effects were statistically reliable despite this limitation, we conducted post hoc power analyses using the *simr* package in R (10,000 iterations). For heart rate, the analysis indicated a very high statistical power of 100.00% (95% CI: 99.96%–100.00%) to detect the effect of the number of consecutive successes at an alpha level of 0.05. Similarly, for performance, the power analysis revealed a statistical power of 99.91% (95% CI: 99.83%–99.96%) to detect the same effect. These results suggest that, despite the limited sample size, the data structure was sufficient to reliably detect relationships between the number of consecutive successes and both heart rate and performance.

## Resource availability

### Lead contact

Requests for further information and resources should be directed to and will be fulfilled by the lead contact, Kagari Yamada (kagari-marines@g.ecc.u-tokyo.ac.jp).

### Materials availability

This study did not generate new unique reagents.

### Data and code availability

• The original data, analysis code, and materials for this study have been deposited in the Open Science Framework (OSF) and are publicly available at https://osf.io/e5kfq/. Accession numbers are listed in the key resources table.
• Any additional information required to reanalyze the data reported in this paper is available from the lead contact upon request.

## Acknowledgments

This study was supported by a Grant-in-Aid for Scientific Research (B) (24K02825) and a Grant-in-Aid for Transformative Research Areas (A) (25H01237) from the Japan Society for the Promotion of Science (10.13039/501100001691JSPS) and by research funds from Kubota Co., Ltd. awarded to K.K. K.Y. was supported by 10.13039/501100001691JSPS as a JSPS Research Fellow.

## Author contributions

Conceptualization and methodology, K.Y., K.M., and K.K; investigation, formal analysis, data curation, visualization, and writing – original draft, K.Y.; writing—review & editing, K.M. and K.K; funding acquisition, resources, and supervision, K.K. All authors read and approved the final manuscript.

## Declaration of interests

The authors declare no competing interests.

## Declaration of generative AI and AI-assisted technologies in the writing process

During the preparation of this work the authors used Gemini (Google) in order to improve the readability and language of the manuscript. After using this tool/service, the authors reviewed and edited the content as needed and take full responsibility for the content of the published article.

## STAR★Methods

### Key resources table

**Table:** 

REAGENT or RESOURCE	SOURCE	IDENTIFIER
**Deposited data**		

Raw data and analysis code	OSF	https://osf.io/e5kfq/.

**Software and algorithms**		

MATLAB	The Math Works, Inc.	https://www.mathworks.com
EMGworks Acquisition system	Delsys	https://delsys.com/

### Experimental model and study participant details

Fifteen male participants (mean age ± SD = 22.5 ± 3.52 years; age range: 19–32 years) took part in Experiment 1. A separate group of fifteen male participants (mean age ± SD = 19.5 ± 1.75 years; age range: 18–25 years) took part in Experiment 2; none had participated in Experiment 1. Participants were limited to males because the experimenter responsible for attaching ECG electrodes to the chest was male; this decision was made to respect participant privacy and comfort during the procedure. One participant in Experiment 1 and one in Experiment 2 self-reported as left-handed. All participants in both experiments reported normal or corrected-to-normal vision. None reported a history of cardiac disease.

This study was approved by the Ethical Review Committee for Experimental Research Involving Human Subjects, Graduate School of Arts and Sciences, University of Tokyo (approval number: 928). All experimental procedures were conducted in full accordance with the approved guidelines. All participants provided written informed consent prior to their participation.

### Method details

#### Apparatus

Visual stimuli were controlled using a computer (FUJITSU LIFEBOOK WU2/H1, Windows 11 Pro) and displayed on a monitor (ASUS VG248QE; 1920 × 1080 pixels, 144 Hz refresh rate). Participants were seated in front of a desk with the monitor. They were instructed to place the elbow of their dominant hand on an armrest, their wrist on a box, and their index finger on a pressure sensor (TEAC, TU-QR(T)500N-G). The non-dominant hand and the ipsilateral leg were positioned in a relaxed, resting posture as instructed.

The force exerted by participants was converted to a voltage signal by the pressure sensor, low-pass filtered at 30 Hz using a digital indicator (TEAC, TD-700T), generating a voltage signal where 100 N corresponded to 10 V. This voltage signal was transmitted to a data acquisition device (National Instruments, USB-6218 (BNC)) and digitally recorded at a sampling frequency of 1000 Hz on a computer running MATLAB with the Data Acquisition Toolbox.

Heart rate was measured using an electrocardiographic (ECG) sensor (Delsys, Delsys Trigno EKG Biofeedback Sensor). Two pre-lubricated disposable ECG electrodes (Nihon Kohden, Dispo Electrode M Vitrode) were attached to each participant’s left clavicle and left side of the chest, following the manufacturer’s recommended placement. ECG data were digitally recorded at a sampling frequency of 519 Hz using an EMGworks Acquisition system (Delsys). A trigger (Delsys, Trigger Module SP-U02) connected to the stimulus computer controlled the ECG sensor.

All visual stimuli and experimental control programs were created using MATLAB. The pressure sensor was calibrated for each participant before the experiment.

#### Procedure

After attaching electrodes and ECG sensors, participants adjusted their seating posture. They then performed a brief calibration by pressing the pressure sensor with maximum force for two seconds; the highest value recorded was defined as their maximum force. Next, participants rested silently for three minutes, during which ECG was recorded. The 60–180 s segment was used as resting heart rate data.

Training consisted of 50 trials requiring participants to exert 10% of their maximum force. Each trial included a countdown, a one-second “PUSH” phase, and feedback. The feedback displayed a numerical ratio of actual to target force, with green indicating success (ratio 0.9–1.1) and red with “HIGH” or “LOW” indicating error.

In Experiment 1, each trial began with a 3-second display of the number of consecutive successes, followed by a countdown, a force application phase, and binary feedback (“success” or “failure” with “HIGH” or “LOW”). Each block consisted of a minimum of 100 trials. To ensure that a streak of successes was not artificially terminated by the end of a block, if a participant was on a success streak at the 100th trial, trials continued until a failure occurred. A block therefore only concluded after a failed trial. Participants rested for five minutes between blocks. The experiment ended after either achieving 10 consecutive successes or 90 minutes.

In Experiment 2, the procedure was the same except that the displayed number indicated total successes rather than consecutive ones. The experiment ended after 100 total successes or 90 minutes. All conditions were explained in advance.

The total number of trials for each participant varied depending on their performance and which of the two stopping criteria was met first. The experiment concluded when a participant either achieved the specified goal (10 consecutive successes in Experiment 1; 100 total successes in Experiment 2) or reached the 90-minute time limit. The resulting trial counts for each participant are detailed in Tables S2 and S3.

#### Analysis

The ECG data were converted to CSV format using the Delsys File Utility (Delsys) and analyzed in MATLAB. R-peaks in the ECG signal were identified using the *findpeaks.m* function. To ensure accurate detection, parameters such as the minimum peak prominence were adjusted for each participant based on their individual R-wave amplitude. The intervals between consecutive R-peaks (R-R intervals) were then calculated. First, we calculated the average of the R-wave intervals from the resting ECG data. We then converted this data to heart rate, in order to record the resting heart rate value for each participant. Next, we calculated the heart rate in each trial, and the difference between them and the resting heart rate was obtained as the heart rate data for each trial. To remove the effects of the task outcome, the ECG data when the feedback was displayed were excluded, and the data from the presentation of the number of consecutive successes just before the presentation of the trial results were analyzed. Therefore, the heart rate calculation for each trial incorporates the data from both the motor execution phase and the pre-trial phase, which consisted of a 3-second display of the number of consecutive successes in Experiment 1 or total successes in Experiment 2. Data from 14 trials by one participant were excluded because of the failure to acquire ECG data.

All behavioral data were analyzed using a custom-made MATLAB program. The voltage data sampled at 1000 Hz were normalized relative to the target value. The difference between the maximum value of them and one (i.e., the target value) was obtained as the relative error and the absolute value of the relative error was taken as an index of force error. In our analysis (e.g., Figure 3), the force error for each consecutive success is the error recorded on the single trial immediately following that n-trial streak.

### Quantification and statistical analysis

The experiment was terminated after ten consecutive successes; therefore, the participants executed the task under conditions ranging from zero to nine consecutive successes. The means and standard deviations were computed for each participant in each consecutive success period. However, six participants had a maximum number of consecutive successes of less than 10, so their mean or standard deviation in each condition was computed from zero consecutive successes to their maximum number of consecutive successes. Because the previous trial was a failure, we assumed that factors other than pressure might have influenced the participants; therefore, we excluded the data from 0 consecutive successes from the analysis. In other words, data from 1 to 9 consecutive successes were used in the analysis.

Data analysis was conducted using a linear mixed-effects model (LMM, using MATLAB Statistics and Machine Learning Toolbox). Compared to analysis of variance, LMM is robust even when there are several randomly distributed missing values^57^ and emphasizes individual differences.^58^ In this study, some participants had missing values owing to the experimental paradigm. In addition, physiological responses to pressure have been found to vary among individuals even with similar pressure manipulations.^34^ Therefore, the LMM was chosen for analysis in this study.

The LMM was used to examine the impact of the number of consecutive successes on heart rate and performance data. LMM uses multiple data points per participant rather than averaging across blocks, and therefore allows changes in psychological pressure to be more accurately mapped onto changes in the dependent variables.^59^ We fitted two mixed models to test whether the number of consecutive successes predominantly exhibited a monotonic or non-monotonic (i.e., second-order) effect on the heart rate data and the performance data (y). The fixed effects were specified as:

(Equation 1) $$\begin{array}{c} \;\text{Model}\;1:y\;∼\;\beta_{0}1+\beta_{1}{CS}^{1} \end{array}$$

(Equation 2) $$\begin{array}{c} \;\text{Model}\;2:y\;∼\;\beta_{0}1+\beta_{1}{CS}^{1}+\beta_{2}{CS}^{2} \end{array}$$

where *β* represent the regression coefficients and *CS* denotes the number of consecutive successes. Specifically, *CS*^1^ is the first-order (linear) term for this variable, and *CS*^2^ is the second-order (quadratic) term. The model included both random intercepts and random slopes for each participant. Random intercepts were included to account for baseline individual differences (e.g., in heart rate or performance), while random slopes for the number of consecutive successes were included to account for individual differences in reactivity to the pressure manipulation. The LMM parameters were estimated using maximum likelihood estimation methods. We accepted the default values for the other options. We investigated whether *β*_1_ was significant in Model 1 and whether *β*_2_ was significant in Model 2.

To examine the relationship between the number of consecutive successes and heart rate and performance, we fitted both a linear and an exponential model to the data. Heart rate and performance data were averaged across participants for each level of consecutive successes. Model fitting was performed using *fit.m* function. A linear model and a single-term exponential model were applied to the mean data. The quality of each model fit was evaluated using the coefficient of determination, the Akaike Information Criterion (AIC), and the Bayesian Information Criterion (BIC), calculated based on the residual sum of squares and the number of model parameters.

The significance of the fixed-effects coefficients in the LMMs was evaluated using a t-test with Satterthwaite’s approximation for the degrees of freedom. For all analyses, the significance level was set at α = 0.05.

### Additional resources

This research is not a clinical trial; no clinical registry applies.
