# Supplementary material for: An experimental paradigm to manipulate physiological arousal using consecutive successes
Source: iScience. 2025 Nov 6;28(12):113961. doi: 10.1016/j.isci.2025.113961 (PMC12671344; doi:10.1016/j.isci.2025.113961)
Supplement: Document S1. Figures S1–S6 and Tables S1–S3 [file mmc1.pdf]

iScience, Volume *28*

Supplemental information

An experimental paradigm to manipulate physiological arousal using consecutive successes

Kagari Yamada, Kohei Miyata, and Kazutoshi Kudo

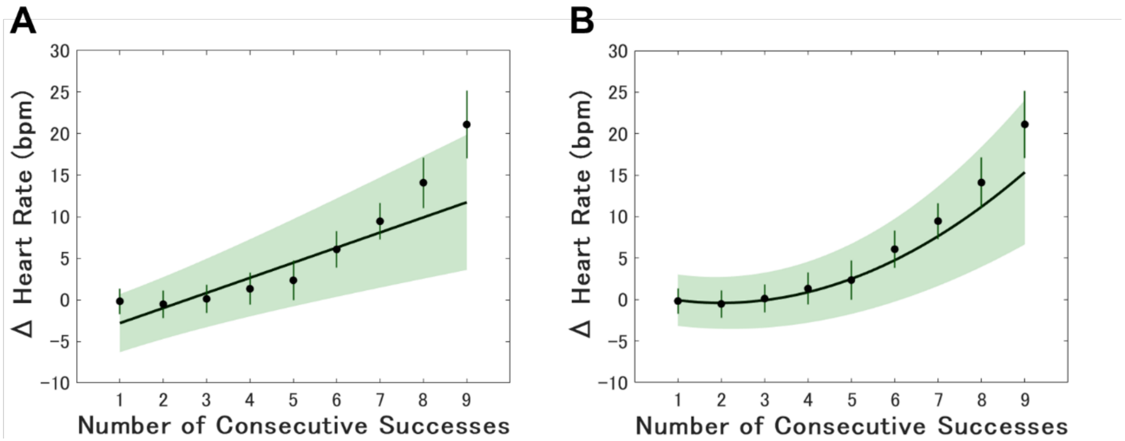

Figure S1. Linear and quadratic mixed-effects models of heart rate in Experiment 1. Heart rate increased significantly with the number of consecutive successes (linear term $\beta = 1.82$, 95% CI [0.89, 2.74], $p < 0.001$) and also showed a significant positive quadratic effect ($\beta = 0.32$, 95% CI [0.20, 0.44], $p < 0.001$), indicating accelerating arousal as streaks grew. (A) Fit of the linear mixed-effects model; the black line shows the fixed-effect estimate and the shaded area the 95% CI. (B) Fit of the quadratic model; the black curve shows the fixed-effect estimate with the 95% CI. In both panels, green dots denote the mean heart rate at each level of consecutive success, and vertical bars represent between-participant standard error.

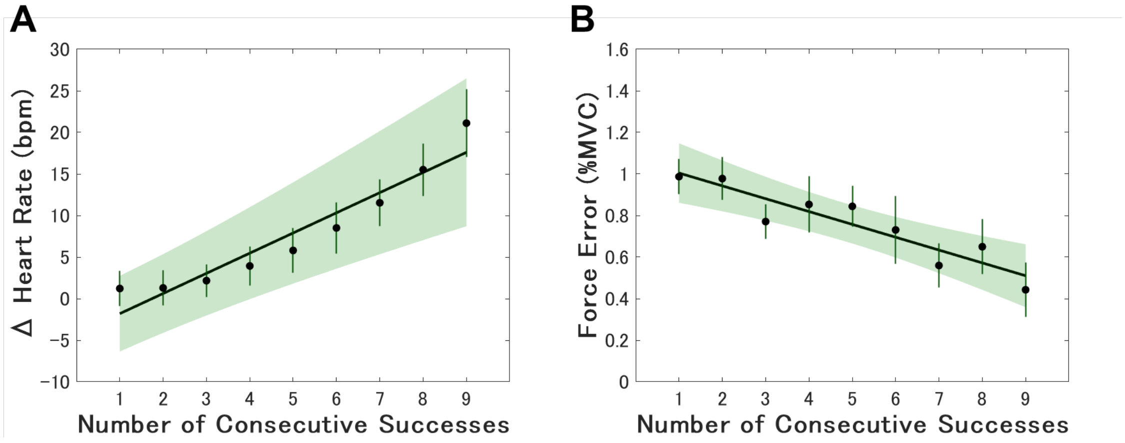

Figure S2. Heart-rate and performance as a function of consecutive successes for participants who achieved 10 in a row (Experiment 1).
Even when restricting analyses to participants who reached nine consecutive successes, heart rate increased linearly with consecutive successes ($\beta = 2.42$, 95% CI [1.41, 3.44], $p < 0.001$), and force error decreased linearly ($\beta = -0.062$, 95% CI [−0.091, −0.032], $p < 0.001$), replicating the main effects and supporting the effectiveness of the

manipulation. (A) Heart-rate difference from resting; (B) force error. Linear regression lines are shown because the fixed-effects coefficients are significant (shaded areas show CIs). Vertical bars represent between-participant standard error.

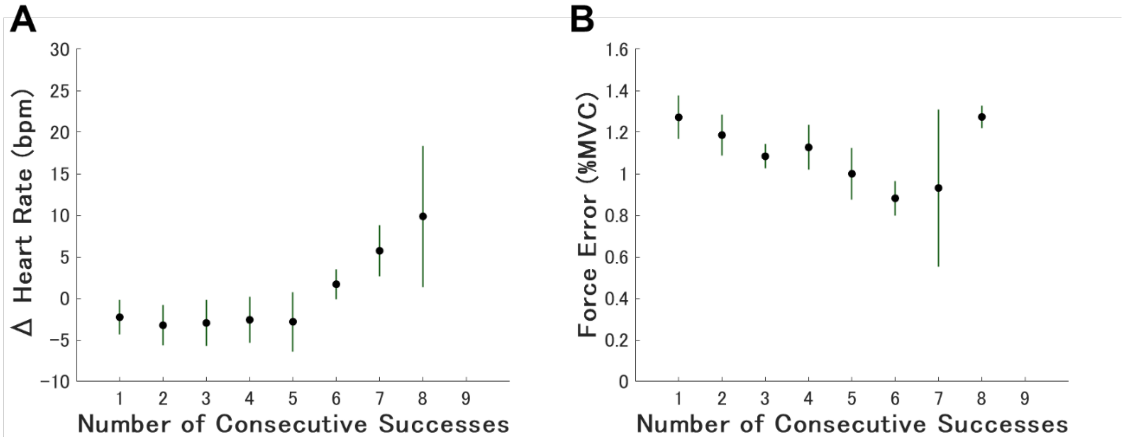

Figure S3. Heart-rate and performance as a function of consecutive successes for participants who did not achieve 10 in a row (Experiment 1).

For participants who did not reach ten consecutive successes, the effect of consecutive successes was not statistically significant for heart rate ($\beta = 0.85$, 95% CI [−0.90, 2.59], $p = 0.28$) or for force error ($\beta = -0.033$, 95% CI [−0.083, 0.017], $p = 0.19$). Despite the small sample and non-significant effects, the estimated coefficients showed trends similar to those from participants who achieved ten. Vertical bars represent between-participant standard error.

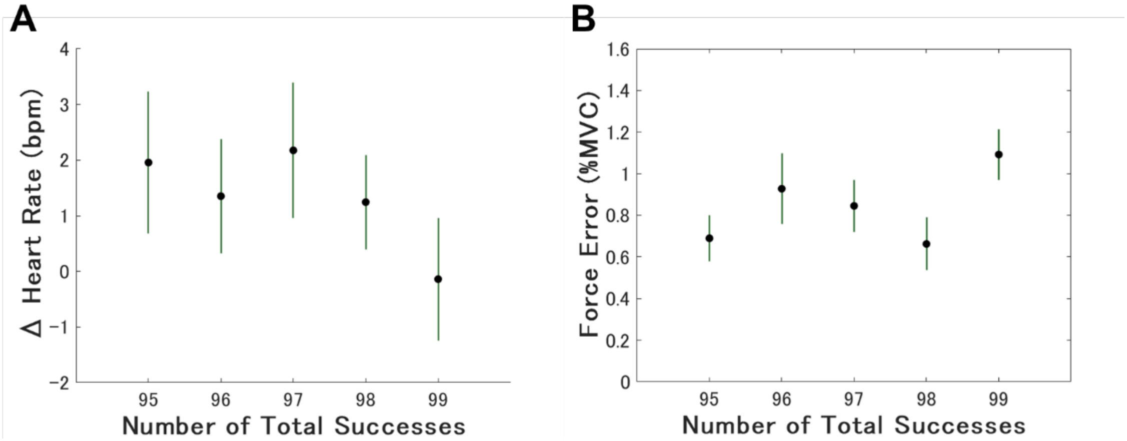

Figure S4. Heart rate and performance as a function of total successes when approaching the goal in Experiment 2.

Approaching the 100-success goal did not increase heart rate ($\beta = -0.054$, 95% CI

[−1.23, 0.15], $p = 0.12$) and did not improve performance (force error: $\beta = 0.031$, 95% CI [−0.080, 0.14], $p = 0.58$), suggesting that the effects in Experiment 1 were not driven by simple goal proximity. (A) Heart-rate difference from resting for total successes 95–99. (B) Force error for total successes 95–99. Vertical bars represent between-participant standard error.

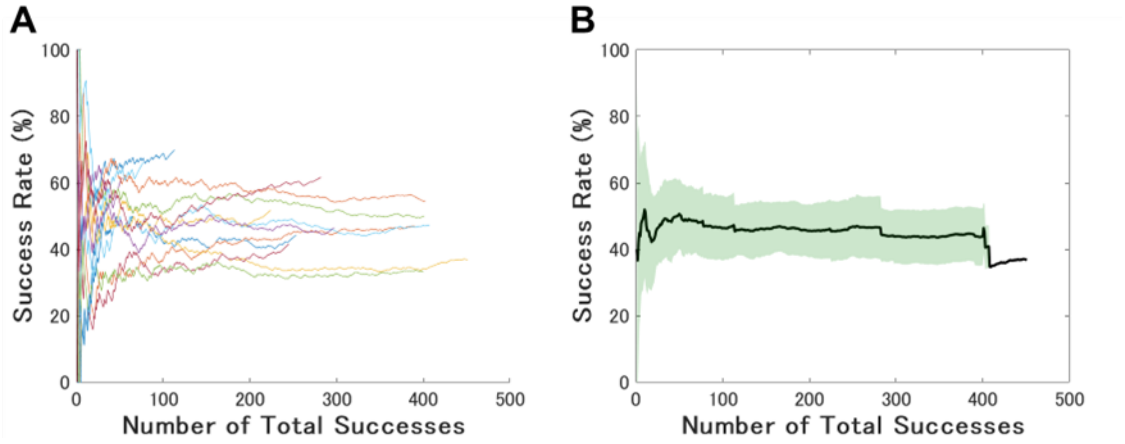

Figure S5. Change in success rate across trials in Experiment 1.
Success-rate trajectories showed minimal systematic learning during the session, consistent with pressure increasing as longer success streaks happened by chance. (A) Success-rate change for each participant. (B) Grand average across participants (black lines) with standard deviation (green shading).

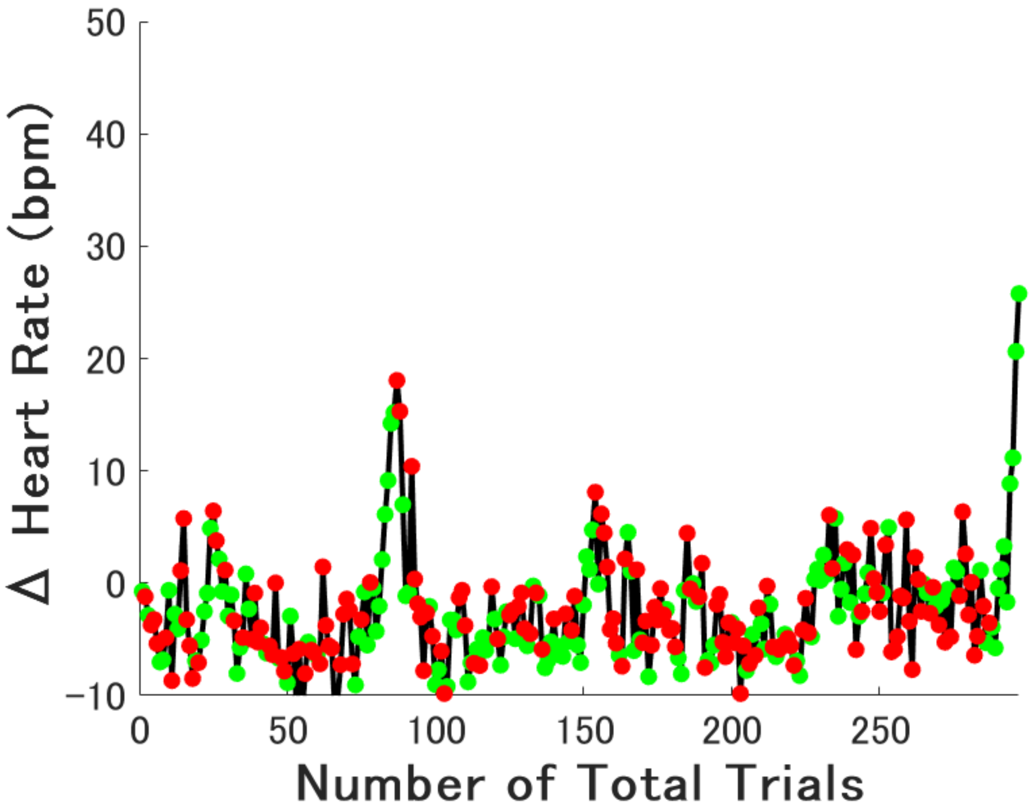

Figure S6. Representative heart rate dynamics across all trials for a single participant in Experiment 1. The black line indicates the trial-by-trial change in heart rate from baseline. Green dots represent successful trials, and red dots represent failed trials. The data illustrate that heart rate tends to ramp up during consecutive successes (e.g., trials 80-90) and gradually decays toward baseline after a failed trial, rather than resetting immediately.

Table S1. Increased heart rate induced by psychological pressure in previous studies.

Study	HR Difference (bpm)	How to manipulate pressure						
		Evaluation	Recording	Competition	Hights	Time	Money	Audience
Yoshie et al., 2009	34.2	○		○			○	○
Nieuwenhuys et al., 2008	13.3				○			
Furuya et al., 2021	12.4	○						
Hasegawa et al., 2013	11.4						○	○
Modi et al., 2018 (Senior Groop)	11.00					○		
Oudejans & Pijpers, 2009	10.0	○	○				○	
Tanaka & Sekiya, 2010 (Experts Groop)	9.86						○	○
Tanaka & Sekiya, 2010 (Novices Groop)	9.86						○	○
Tanaka & Sekiya, 2011	8.31						○	
Sasaki & Sekiya, 2018	7	○						
Oudejans & Pijpers, 2010	6.9	○	○				○	
Englert et al., 2015	5.6				○			
Gorgulu et al., 2019 (Experiment 5)	5.20			○			○	
Yoshie et al., 2008	5.0	○						
Gorgulu et al., 2019 (Experiment 4)	4.77			○			○	
Gorgulu & Gokcek, 2021	4.66			○			○	
Modi et al., 2018 (Intermediate Groop)	4.00					○		
Cooke et al., 2014	3.88			○			○	
Gorgulu et al., 2019 (Experiment 3)	3.87			○			○	
Sekiya & Tanaka, 2019	3.46						○	
Cooke et al., 2010	3.13		○	○			○	
Ogawa & Sekiya, 2016	2.79	○	○				○	
Oudejans et al., 2013	2.73				○			
Gorgulu et al., 2019 (Experiment 1)	2.59			○			○	
Klämpfl et al., 2013	2.4		○				○	
Gorgulu et al., 2019 (Experiment 2)	2.22			○			○	
Daou et al., 2019	1.8	○	○				○	
Malhorta et al., 2012	1.12					○		
Modi et al., 2018 (Junior Groop)	-1.00					○		

We listed studies that manipulated psychological pressure. The “HR Difference (bpm)” is the difference in mean heart rate between conditions with and without pressure. The right side of the table shows the method used by experimenters to induce pressure on participants.

Table S2. Number of trials in each consecutive success for each participant in Experiment 1

	0	1	2	3	4	5	6	7	8	9	Total
1	142	52	23	13	10	5	3	2	2	1	253
2	207	90	50	25	11	4	2	1	0	0	390
3	285	98	37	14	6	4	3	2	2	0	451
4	21	12	7	4	2	2	1	1	1	1	52
5	205	99	49	21	14	5	4	3	1	0	401
6	215	92	46	24	13	7	5	3	2	0	407
7	144	48	22	10	9	5	4	1	1	1	245
8	35	25	19	14	8	4	3	2	2	1	113
9	183	91	53	38	21	10	4	2	0	0	402

10	109	50	30	15	9	4	2	2	2	1	224
11	160	69	33	15	7	4	4	2	2	1	297
12	267	84	29	13	6	1	0	0	0	0	400
13	27	16	11	9	5	3	2	2	1	1	77
14	109	53	35	28	18	12	10	9	5	3	282
15	17	7	4	1	1	1	1	1	1	1	35
Ave.	141.7	59.1	29.9	16.3	9.3	4.7	3.2	2.2	1.5	0.7	268.6

Note. Each column shows the number of trials in each consecutive success, and each row shows the number of trials for each participant.

Table S3. Number of trials in each consecutive success for each participant in Experiment 2.

	0	1	2	3	4	5	6	7	8	9	Total
1	90	41	24	17	8	4	3	2	0	0	189
2	224	65	22	8	2	2	0	0	0	0	323
3	78	42	23	13	10	5	3	3	0	0	177
4	116	53	23	12	6	1	1	1	1	1	215
5	221	54	17	6	3	0	0	0	0	0	301
6	79	45	25	13	7	4	2	2	1	0	178
7	56	38	22	15	9	6	4	3	1	1	155
8	77	41	23	14	7	3	2	2	1	1	176
9	80	40	22	16	10	6	2	1	1	1	179
10	122	49	22	13	7	4	2	2	0	0	221
11	67	32	23	15	8	7	5	4	3	1	166
12	98	45	25	12	6	4	3	2	1	1	197
13	142	53	24	14	5	3	0	0	0	0	241
14	68	48	24	10	7	3	2	2	1	1	167
15	104	48	28	11	5	4	2	1	0	0	203
Ave.	108.1	46.3	23.1	12.6	6.7	3.7	2.1	1.7	0.7	0.5	205.9

Note. Each column shows the number of trials in each consecutive success, and each row shows the number of trials for each participant.
